# Supplementary material for: “It Empowers You to Empower Them”: Health Professional Perspectives of Care for Hyperglycaemia in Pregnancy Following a Multi-Component Health Systems Intervention
Source: Int J Environ Res Public Health. 2024 Aug 28;21(9):1139. doi: 10.3390/ijerph21091139 (PMC11431348; doi:10.3390/ijerph21091139)
Supplement: Supplementary file 1 [file ijerph-21-01139-s001.zip › ijerph-3113590-supplementary.pdf]

Supplementary

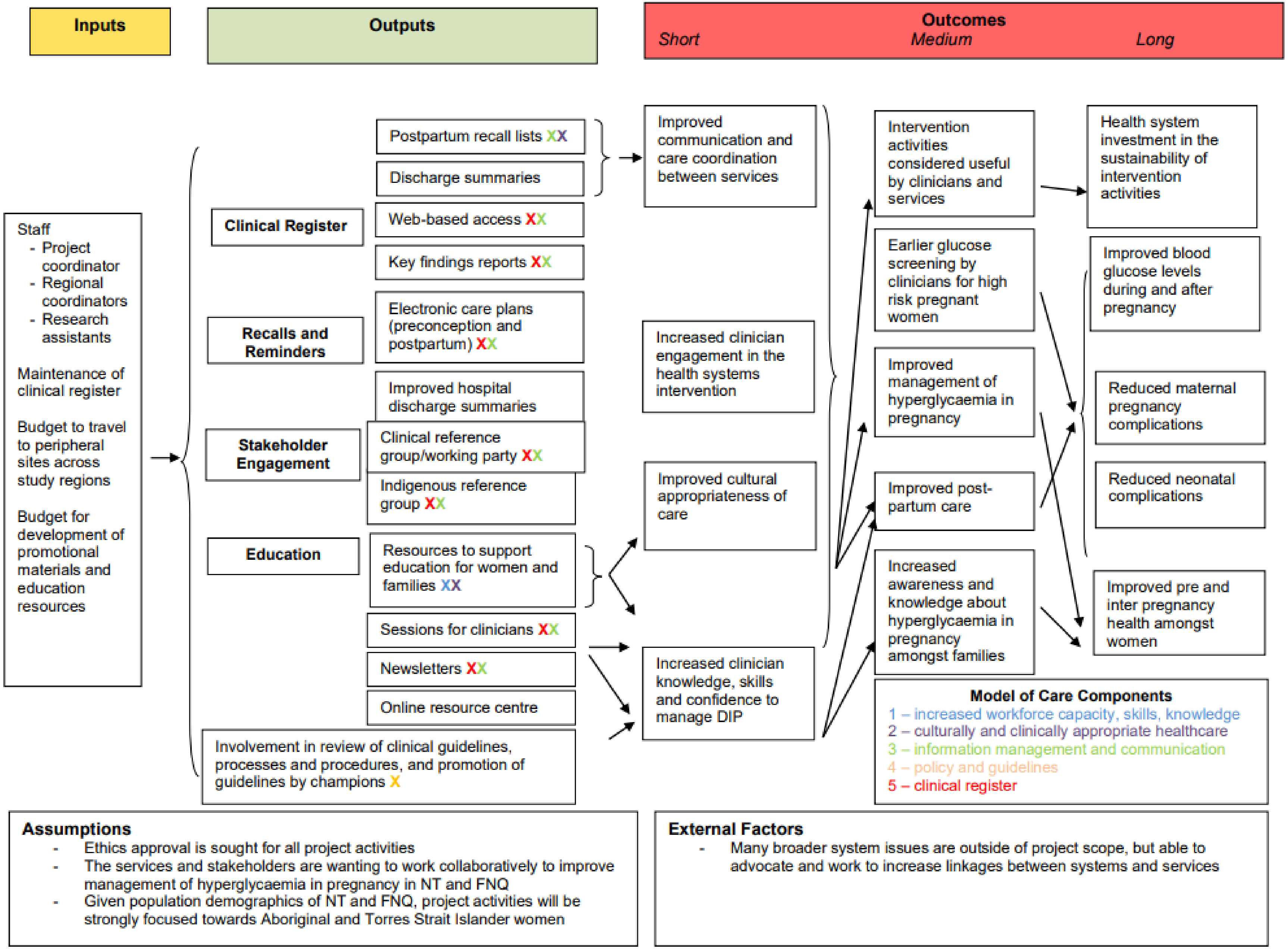

Figure S1 – Logic model for a health systems intervention to improve care during and after a pregnancy complicated by hyperglycaemia (licensed under CC BY 4.0 by MacKay D et al[25])

Table S1 – Implementation activities to improve care for women during and after a pregnancy complicated by hyperglycaemia						
**Activity**	**Models of Care Components**	**Procedure**	**Materials**	**Delivered by**	**Mode of delivery**	**Region (TE, CA, FNQ, All)**
**Education for healthcare providers**	1, 2, 3, 4	Develop an education calendar across each region to plan and deliver educational activities to healthcare practitioners, aligning with educational activities of other regional healthcare organisations (e.g. primary care networks) where possible, with invitation of healthcare providers through healthcare networks<br>Selected sessions will be recorded and delivered online to enable access for practitioners unable to attend, with information for access distributed and promoted through Partnership networks and health practitioner organisations<br>Newsletters to be distributed to healthcare providers and stakeholders through Partnership networks<br>Annual symposium, with healthcare providers and other stakeholders invited through Partnership networks	Presentations<br>Newsletters<br>Workshops<br>Online resources (videos, podcasts)	Educational materials, including presentations and text-based materials, developed by project staff, with input from clinical experts (endocrinologists, diabetes nurse practitioner and educators, primary care practitioners) and Indigenous reference group<br>Education sessions delivered by clinicians and project staff	Face-to-face<br>Online<br>Email<br>Teleconference	All
**Postpartum care plans and reminders**	2, 3, 5	Develop postpartum care plans and reminders to align with and bridge to existing Chronic Disease Care Plan after pregnancy and embed within primary healthcare electronic health record, to prompt healthcare provider recall of women at recommended timepoints for review based on guidelines	Electronic primary care health record	Implementation team in collaboration with health services staff, with input from clinical reference group	Electronic primary care health record	TE, CA
**Preconception care plans**	2, 3, 5	Develop pre-conception care plans and embed within primary healthcare electronic health record, to prompt healthcare provider to ensure recommended pre-conception care is delivered to women with pre-existing diabetes based on guidelines	Electronic primary care health record	PhD student with input from clinical reference group	Electronic primary care health record	CA
**Indigenous reference group**	2, 3	Form an Indigenous reference group to provide input regarding priority-setting, resource development and implementation, by inviting Indigenous women with an interest in hyperglycaemia in pregnancy, meeting three times per year and feeding back to investigators and project staff	Advice from Director of Aboriginal Programs, Menzies School of Health Research	Coordinated by Indigenous implementation team member	Face-to-face, with email communication between meetings	TE, CA
**Clinical reference group**	2, 3	Ongoing facilitation of a reference group of clinicians to provide input regarding priority-setting, resource development and implementation, by inviting interested clinicians through Partnership networks, to meet annually and feed back to investigators and project staff	Presentations; meetings; circulation of documents/ resources for comment	Coordinated by implementation team	Face-to-face, with clinicians provided with email address to provide feedback between meetings	TE, CA
**Working group**	2, 3	Form a working group with representatives from partner organisations to provide input regarding priority-setting, resource development and implementation, as well as opportunity for promotion of educational opportunities	Presentations; meetings; circulation of documents/ resources for comment	Coordinated by implementation team	Face-to-face meetings alternate months	FNQ

**Resource development**	1, 2, 3	Development of culturally appropriate resources to assist healthcare providers in discussions with women about health after a pregnancy complicated by diabetes	Postpartum discharge brochure	Discharge brochure developed by implementation team with input from clinical experts	Paper-based	All
**Aggregate DIP Clinical Register reports**	1, 3, 5	Produce de-identified aggregate postpartum reports from the DIP Clinical Register six-monthly and distribute to healthcare providers and stakeholders to enable quality improvement activities	DIP Clinical Register	Implementation team	Email	All
**Local DIP Clinical Register reports**	1, 3, 5	Produce local postpartum reports with identifiable data from the DIP Clinical Register six-monthly and distribute to healthcare providers to aid in quality improvement activities and recall of women	DIP Clinical Register	Implementation team	Email	TE, CA
**Modified discharge summaries**	3, 5	Review and amend current discharge summary templates for discharge from hospital after delivery, to include options and prompts to facilitate communication of follow-up plans between hospital and primary care providers	Discharge summaries	Implementation team	Within electronic discharge summary	TE, CA
**Postpartum summary**	3, 5	Generate postpartum diabetes in pregnancy summary using DIP Clinical Register data and distribute to healthcare providers, including reminder for postpartum screening	DIP Clinical Register	Implementation team	Letter	FNQ
**Postpartum screening reminder letters**	3, 5	Generate letters to healthcare providers using DIP Clinical Register data to prompt recall of women for recommended postpartum glucose check if check not recorded within six months postpartum	DIP Clinical Register	Implementation team	Letter	FNQ
**Promotion of postpartum guidelines by champions**	1, 4	Champions identified through engagement with Partnership activities, and upskilled regarding use of local guidelines through Partnership educational activities and publications	Local clinical guidelines – CARPA (CA, TE), QCG (FNQ)	Implementation team	Face-to-face, email	All

Models of Care Components: 1 – Increasing workforce capacity, skills and knowledge and improvement in the health literacy of health professionals and women; 2 – Improving access to culturally and clinically appropriate healthcare; 3 – Improving information management and communication; 4 – Enhancing policy and guidelines; 5 – Embedding the Diabetes in Pregnancy Clinical Register as a component with the Models of Care
Abbreviations: CA - Central Australia, CARPA – Central Australian Rural Practitioners Association (2017), DIP – Diabetes in Pregnancy, FNQ - Far North Queensland, QCG – Queensland Clinical Guidelines (2015), TE - Top End, the Partnership – Diabetes Across the Lifecourse: Northern Australia Partnership

Supplementary Table is a derivative from the original work licensed under CC BY 4.0 by MacKay D et al

Table S2 – Indicators for mixed-methods evaluation of a multi-component health systems intervention to improve antenatal and postpartum care for hyperglycaemia in pregnancy				
**Objective**	Final evaluation question addressed*	Indicator	Data source	Data collection
		REACH		
**Increase (FNQ) and sustain (NT) engagement of clinicians with the project**	3<br>3<br>3	Health practitioner awareness of Partnership and activities<br>Health practitioner attendance at Partnership education events<br>Use of project online health professional educational resources	Health professionals<br>Project activity log<br>Website	Interviews<br>Surveys<br>Activity log<br>Metrics from website
**Improve health practitioner awareness of DIP Clinical Register**	3	Health practitioner awareness of DIP Clinical Register	Health professionals<br>DIP Clinical Register	Interviews<br>Surveys
**Increase (FNQ) and sustain (NT) coverage of the DIP Clinical Register**	1	DIP Clinical Register coverage; trajectory of coverage over time	Comparison of DIP Clinical Register with health service data	Health service reports
**Determine the number and characteristics of women accessing and not accessing antenatal care**		Number of women accessing antenatal care, including number and timing of visits	Health service electronic health records<br>DIP Clinical Register<br>Health service reports[1]	Audit
		EFFECTIVENESS		
**Enhance support for health practitioners**	1<br>4	Health practitioner perception of support<br>Health practitioner and champion reports of which activities have been useful in enhancing support	Health professionals<br>Champions	Interviews<br>Surveys
**Increase health practitioner awareness of and confidence in managing hyperglycaemia in pregnancy**	1<br><br>1	Health practitioners perceived knowledge and confidence, and changes from Partnership formative work<br>Rates of completion of recommended glucose screening in early pregnancy for high risk women	Health professionals<br>Health service electronic health records<br>DIP Clinical Register<br>Formative DIP Models of Care work	Interviews<br>Surveys<br>Audit
**Earlier hyperglycaemia in pregnancy screening women at high risk**	1	Rates of completion of recommended early pregnancy screening for high risk women	DIP Clinical Register	Audit
**Improved blood glucose levels for women with diabetes in pregnancy**	2	Mean first- and third-trimester HbA1c and changes over time	DIP Clinical Register	Audit
**Improved birth and neonatal outcomes**	2	Gestational age at delivery<br>Mode of delivery<br>Birth weight<br>Large for gestational age<br>Small for gestational age<br>Macrosomia<br>Neonatal obstetric trauma<br>Neonatal hypoglycaemia requiring treatment<br>Neonatal special care admission<br>5-minute APGAR score less than 5<br>Neonatal jaundice requiring treatment<br>Neonatal respiratory distress	DIP Clinical Register	Audit
**Improve health practitioners' awareness of postpartum guidelines**	1	Health practitioner awareness of guidelines and changes over time	Health professionals<br>Champions	Interviews<br>Surveys

**Improve postpartum management, according to guidelines, following diabetes in pregnancy**	2<br>2<br>2<br>2<br>2<br><br>2	Proportion of women completing postpartum glucose testing<br>Postpartum weight, body mass index, waist circumference<br>Proportion of women breastfeeding<br>Proportion of women smoking<br>Proportion of women prescribed contraception, or who have discussed contraception with a health practitioner<br>Changes over time in all indicators	Electronic health records<br>DIP Clinical Register	Audit
**Enhance communication between primary healthcare and hospital services**	1<br><br>3	Health practitioner perception of communication between primary healthcare and hospital services<br>Health practitioner and champion reports of which activities have contributed to changes	Health professionals<br>Champions	Interviews<br>Surveys
**Improve referral pathways and care coordination for services caring for women with hyperglycaemia in pregnancy**	1<br>1<br>3	Health practitioner knowledge of referral pathways<br>Health practitioner perception of improvements in care coordination<br>Health practitioner and champion reports of which activities have contributed to changes	Health professionals<br>Champions	Interviews<br>Surveys
**Improve discharge processes**	1<br>1	Health practitioner perception of usefulness of discharge summaries<br>Health practitioner and champion perception of impact of discharge processes on postpartum care	Health professionals<br>Champions	Interviews<br>Surveys
		ADOPTION		
**Enhance referrals to DIP Clinical Register**	1<br>3	DIP Clinical Register coverage<br>Health service perceptions of referral process	Comparison of DIP Clinical Register with health service data<br>Health professionals	Health service reports[1]<br>Interviews<br>Surveys
**Improve practitioner use of DIP Clinical Register**	1<br>3	Health practitioner use of DIP Clinical Register and reports<br>Health practitioner reports of which aspects of reports are useful in practice	Health professionals<br>DIP Clinical Register	Interviews<br>Surveys<br>External use of DIP Clinical Register website, e.g. website metrics
**Identify enablers and barriers impacting on adoption of project activities**	3	Health practitioner, implementer and champion reports of enablers and barriers	Health professionals<br>Implementers<br>Champions	Interviews
**Determine acceptability and value of project activities**<br>• **Are project activities socially appropriate/ acceptable?**<br>• **What is the social importance of project outcomes?**<br>• **Which project activities are perceived as valuable?**	4	Health practitioner, implementer and champion perceptions of acceptability and value of project activities	Health professionals<br>Implementers<br>Champions<br>Women	Interviews
		IMPLEMENTATION		
**Determine if project activities have been delivered as intended**	3	Proportion of planned activities delivered	Project activity log<br>Implementers	Interviews<br>Audit of activity log

**Determine if project activities have been adapted, e.g. to fit local needs**	3	Adaptations of planned activities and rationale	Implementers	Interviews
**Identify enablers and barriers impacting on implementation of project activities**	3	Enablers and barriers as identified by implementation team	Implementers<br>Health professionals<br>Champions	Interviews
		MAINTENANCE		
**Sustain DIP Clinical Register through integration with other structures**	5<br><br>5	Health practitioners and services perceptions of sustainability of the DIP Clinical Register<br>Resources required and cost of maintaining DIP Clinical Register	Health professionals<br>Champions<br>Implementers<br>Activity log	Interviews<br>Surveys<br>Cost-consequences analysis
**Identify project activities sustainable beyond project completion, and method for funding or integration into existing services**	5<br><br>5	Health professional, champion and implementer perception of sustainability of project activities<br>Resources required for project activity sustainability	Health professionals<br>Champions<br>Implementers<br>Activity log	Interviews<br>Cost-consequences analysis
**Supplementary 2: Table is a derivative from the original work licensed under CC BY 4.0 by MacKay D et al**				

Additional Methods

Survey Methods

Health professional survey topics included: health professional confidence and knowledge regarding hyperglycaemia in pregnancy; health professional's usual practice, including screening and managing hyperglycaemia in pregnancy; use of guidelines and patient education resources; and satisfaction with care pathways and communication between services. Surveys were piloted by clinicians, with modification for question clarity made based on feedback from piloting.

Surveys were distributed online and in hard copy through Partnership mailing lists and distributed by partner organisations (government and non-government health services at primary, secondary and tertiary care levels) to relevant staff. Online participants were asked to forward the survey link through their networks. Hard copies were available at relevant meetings and workshops, and supplied to partner organisations. Due to the high staff turnover in the NT and FNQ, participants were recruited independently for the baseline and evaluation surveys, i.e. responses for each survey are not linked to respondents.

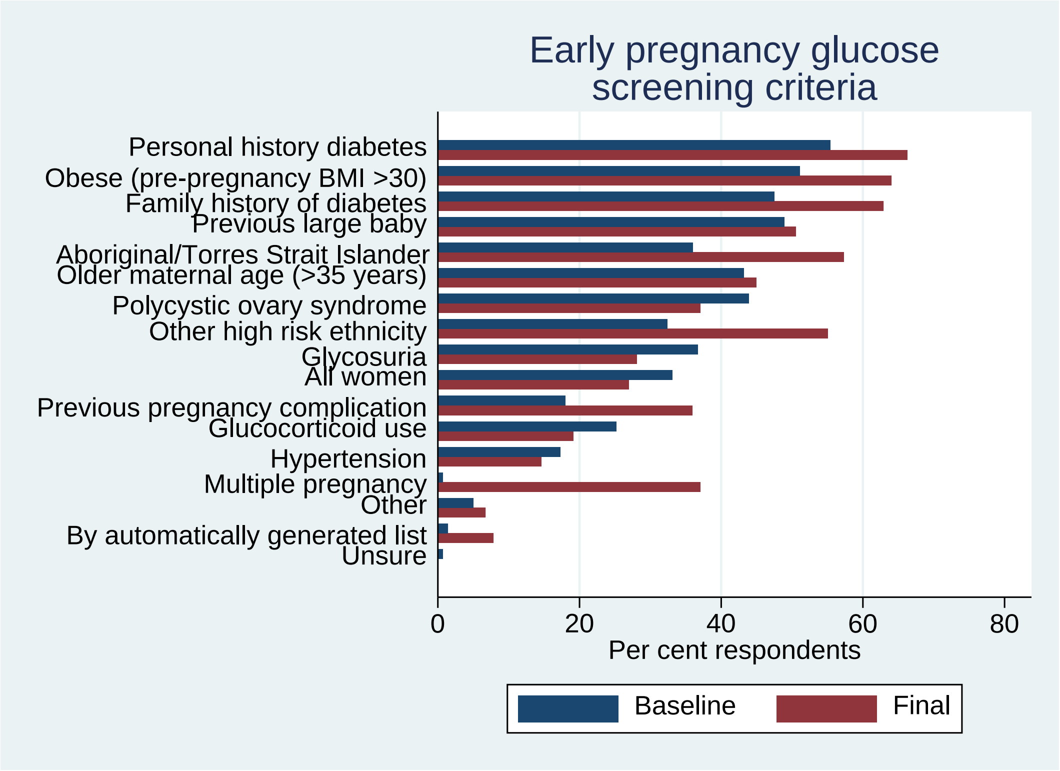

Figure S2 – Criteria used to select women for glucose screening in early pregnancy

Table S3 Survey results by region

Participant characteristics								
	All regions		Central Australia		Far North Queensland		Top End	
	Baseline N=183 (%)	Post-intervention N=137 (%)	Baseline N=36 (%)	Final N=40 (%)	Baseline N=101 (%)	Final N=52 (%)	Baseline N=45 (%)	Final N=45 (%)
Occupation								
Nurse	9 (4.9)	5 (3.6)	3 (8.3)	4 (10.0)	1 (1.0)	1 (1.9)	2 (4.4)	0 (0.0)
Midwife	57 (31.1)	67 (48.9)	9 (25.0)	17 (42.5)	37 (36.6)	21 (40.4)	10 (22.2)	29 (64.4)
General Practitioner/GP Obstetrician	38 (20.8)	10 (7.3)	9 (25.0)	3 (7.5)	17 (16.8)	4 (7.7)	11 (24.4)	8 (5.8)
Medical Specialist	9 (4.9)	5 (3.6)	2 (5.6)	2 (5.0)	7 (6.9)	3 (5.8)	0 (0.0)	0 (0.0)
Medical Practitioner (other)	3 (1.6)	1 (0.7)	0 (0.0)	1 (2.5)	3 (3.0)	0 (0.0)	0 (0.0)	0 (0.0)
Diabetes Educator	23 (12.6)	25 (18.2)	4 (11.1)	4 (10.0)	12 (11.9)	12 (23.1)	7 (15.6)	9 (20.0)
Aboriginal Health Practitioner/Worker	12 (6.6)	3 (2.2)	1 (2.8)	0 (0.0)	10 (9.9)	1 (1.9)	1 (2.2)	2 (4.4)
Dietitian	12 (6.6)	11 (8.0)	4 (11.1)	6 (15.0)	3 (3.0)	5 (9.6)	5 (11.1)	0 (0.0)
Other	17 (9.3)	10 (7.3)	4 (11.1)	3 (7.5)	9 (8.9)	4 (7.7)	8 (17.8)	4 (8.9)
Region								
Northern Territory – Central Australia	36 (19.8)	40 (29.2)						
Northern Territory – Top End	45 (24.7)	52 (38.0)	N/A	N/A	N/A	N/A	N/A	N/A
Far North Queensland	101 (55.5)	45 (32.8)						
Main work setting								
Regional/remote	117 (67.6)	73 (53.3)	19 (55.9)	21 (52.5)	71 (75.5)	32 (61.5)	26 (59.1)	20 (44.4)
Urban	56 (32.4)	64 (46.7)	15 (44.1)	19 (47.5)	23 (24.5)	20 (38.5)	18 (40.9)	25 (55.6)
Time in current position								
<1 year	33 (18.1)	15 (11.0)	9 (25.0)	7 (18.0)	10 (10.0)	4 (7.7)	14 (31.1)	15 (11.0)
1-5 years	68 (37.4)	54 (39.7)	12 (33.3)	14 (35.9)	44 (44.0)	13 (25.0)	12 (26.7)	54 (39.7)
5-10 years	32 (17.6)	38 (27.9)	7 (19.4)	9 (23.1)	18 (18.0)	23 (44.2)	7 (15.6)	38 (27.9)
>10 years	49 (26.9)	29 (21.3)	8 (22.2)	9 (23.1)	28 (28.0)	12 (23.1)	12 (26.7)	29 (21.3)
Client ethnicity								
Predominantly Aboriginal and/or Torres Strait Islander	76 (42.7)	72 (52.6)	24 (68.6)	33 (82.5)	28 (28.0)	13 (25.0)	23 (54.7)	26 (57.8)
Predominantly non-Indigenous	12 (6.7)	17 (12.4)	4 (11.4)	0 (0.0)	4 (4.0)	16 (30.8)	4 (9.5)	1 (2.2)
Mixed	90 (50.6)	48 (35.0)	7 (20.0)	7 (17.5)	68 (68.0)	23 (44.2)	15 (35.7)	18 (40.0)
Previous participation in DIP survey								
Yes	N/A	23 (16.8)	N/A	6 (15.0)	N/A	12 (23.1)	N/A	5 (11.1)
No		75 (54.7)		21 (52.5)		26 (50.0)		28 (62.2)

Unsure		39 (28.5)		13 (32.5)		14 (26.9)		12 (26.7)
Primary place of work in primary health care								
Yes	143 (78.1)	104 (76.5)	32 (88.9)	33 (82.5)	72 (72.3)	32 (62.8)	37 (84.1)	39 (86.7)
No	40 (21.9)	32 (23.5)	4 (11.1)	7 (17.5)	29 (28.7)	19 (37.3)	7 (15.9)	6 (13.3)
**Participant practice**								
	All regions		Central Australia		Far North Queensland		Top End	
	Baseline N (%)	Final N (%)	Baseline N (%)	Final N (%)	Baseline N (%)	Final N (%)	Baseline N (%)	Final N (%)
Practitioners screening for diabetes in early pregnancy[#]								
Yes	95 (67.9)	81 (81.0)	20 (74.1)	26 (86.7)	54 (65.1)	26 (76.5)	20 (69.0)	29 (80.6)
No	32 (22.9)	1 (1.0)	5 (18.5)	1 (3.3)	23 (27.7)	0 (0.0)	4 (13.8)	0 (0.0)
Unsure	13 (9.3)	1 (1.0)	2 (7.4)	0 (0.0)	6 (7.2)	0 (0.0)	5 (17.2)	1 (2.8)
N/A – not viewed as part of practitioners' role^	N/A	17 (17.0)	N/A	3 (10.0)	N/A	8 (23.5)	N/A	6 (16.7)
Screening test most commonly used in first trimester[#]								
75g oral glucose tolerance test	75 (49.7)	56 (73.7)	13 (54.2)	16 (88.9)	49 (52.1)	17 (63.0)	13 (40.6)	23 (74.2)
HbA1c	33 (21.9)	16 (21.1)	6 (25.0)	2 (11.1)	18 (19.2)	7 (25.9)	8 (25.0)	7 (22.6)
Random plasma glucose/blood glucose level	22 (14.6)	1 (1.3)	0 (0.0)	0 (0.0)	18 (19.2)	0 (0.0)	4 (12.5)	1 (3.2)
Fasting plasma glucose/blood glucose level	4 (2.6)	1 (1.3)	0 (0.0)	1 (0.0)	3 (3.2)	1 (3.7)	1 (3.1)	0 (0.0)
50 gram glucose challenge test	3 (2.0)	2 (2.6)	1 (4.2)	0 (0.0)	1 (1.1)	2 (7.4)	1 (3.1)	0 (0.0)
Unsure	14 (9.3)	0 (0.0)	4 (16.7)	0 (0.0)	5 (5.3)	0 (0.0)	5 (15.6)	0 (0.0)
Screening test most commonly used in second/third trimester[#]								
75g oral glucose tolerance test	114 (83.3)	80 (95.2)	19 (82.6)	24 (96.0)	73 (86.9)	25 (96.2)	21 (75.0)	31 (93.9)
HbA1c	9 (6.6)	1 (1.2)	3 (13.0)	0 (0.0)	3 (3.6)	0 (0.0)	3 (10.7)	1 (3.0)
Random plasma glucose/blood glucose level	2 (1.5)	1 (1.2)	0 (0.0)	1 (4.0)	1 (1.2)	0 (0.0)	1 (3.6)	0 (0.0)
Fasting plasma glucose/blood glucose level	1 (0.7)	0 (0.0)	0 (0.0)	0 (0.0)	0 (0.0)	0 (0.0)	1 (3.6)	0 (0.0)
50 gram glucose challenge test	7 (5.1)	2 (2.4)	1 (4.4)	0 (0.0)	6 (7.1)	1 (3.9)	0 (0.0)	1 (3.0)
Unsure	3 (2.2)	0 (0.0)	0 (0.0)	0 (0.0)	1 (1.2)	0 (0.0)	2 (7.1)	0 (0.0)
Gestational age of second/third trimester glucose screening								
<24 weeks	3 (2.2)	2 (2.3)	1 (4.0)	2 (7.4)	1 (1.2)	0 (0.0)	1 (3.9)	0 (0.0)
24-28 weeks	128 (95.5)	81 (93.1)	24 (96.0)	24 (88.9)	79 (96.3)	26 (96.3)	24 (92.3)	31 (93.9)
>28 weeks	2 (1.5)	2 (2.3)	0 (0.0)	1 (3.7)	1 (1.2)	0 (0.0)	1 (3.9)	1 (3.0)
	1 (0.7)	2 (2.3)	0 (0.0)	0 (0.0)	1 (1.2)	1 (3.7)	0 (0.0)	1 (3.0)

Other								
Proportion of women with hyperglycaemia in pregnancy seen postpartum for ongoing clinical care								
0-20%	54 (39.4)	33 (39.3)	7 (28.0)	10 (33.3)	34 (42.0)	14 (46.7)	13 (43.3)	9 (37.5)
21-40%	21 (15.3)	7 (8.3)	9 (36.0)	4 (133)	9 (11.1)	2 (6.7)	3 (10.0)	1 (4.2)
41-60%	18 (13.1)	8 (9.5)	2 (8.0)	2 (6.7)	12 (14.8)	4 (13.3)	4 (13.3)	2 (8.3)
61-80%	19 (13.9)	13 (15.5)	6 (24.0)	3 (10.0)	7 (8.6)	6 (20.0)	6 (20.0)	4 (16.7)
81-100%	25 (18.2)	23 (27.4)	1 (4.0)	11 (36.7)	19 (23.5)	4 (13.3)	4 (13.3)	8 (33.3)
Practitioners screening for diabetes postpartum after gestational diabetes*~								
Yes	39 (79.6)	90 (76.9)	19 (79.2)	28 (90.3)		32 (76.2)	20 (80.0)	30 (68.2)
No	6 (12.2)	1 (0.9)	3 (12.5)	1 (3.2)	N/A	0 (0.0)	3 (12.0)	0 (0.0)
Unsure	4 (8.2)	0 (0.0)	2 (8.3)	0 (0.0)		0 (0.0)	2 (8.0)	0 (0.0)
N/A – not viewed as part of practitioners' role^	N/A	26 (22.2)	N/A	2 (6.5)		10 (23.8)	N/A	14 (31.8)
Use of recalls for postpartum screening*	44 (88.0)	86 (93.5)	22 (96.7)	27 (93.1)	N/A	29 (87.9)	21 (84.0)	30 (100.0)
Timing of postpartum screening after gestational								
diabetes*	26 (53.1)	17 (19.5)	13 (54.2)	9 (34.6)		3 (9.4)	13 (54.2)	5 (17.2)
Up to and including 6 weeks	13 (26.5)	60 (69.0)	7 (29.2)	14 (53.9)	N/A	24 (75.0)	5 (20.8)	22 (75.9)
After 6 weeks, up to and including 12 weeks	8 (16.3)	8 (9.2)	4 (16.7)	3 (11.5)		5 (15.6)	4 (16.7)	0 (0.0)
After 12 weeks, up to and including 6 months	2 (4.1)	2 (2.3)	0 (0.0)	0 (0.0)		0 (0.0)	2 (8.3)	2 (6.9)
After 6 months, up to and including 12 months								
Screening test most commonly used postpartum after gestational diabetes*								
75g oral glucose tolerance test	31 (64.6)	40 (74.1)	17 (73.9)	18 (69.2)		16 (50.0)	13 (54.2)	22 (78.6)
HbA1c	12 (25.0)	11 (20.4)	5 (21.7)	6 (23.1)	N/A	14 (43.8)	7 (29.2)	5 (17.9)
Random plasma glucose/blood glucose level	2 (4.2)	0 (0.0)	0 (0.0)	0 (0.0)		0 (0.0)	2 (8.3)	0 (0.0)
Fasting plasma glucose/blood glucose level	2 (4.2)	0 (0.0)	1 (4.4)	0 (0.0)		1 (3.1)	1 (4.2)	0 (0.0)
Unsure	1 (2.1)	3 (5.6)	0 (0.0)	2 (7.7)		1 (3.1)	1 (4.2)	1 (3.6)
Resuming HbA1c monitoring postpartum in women with pre-existing diabetes*								
Up to and including 12 weeks	43 (78.2)	47 (54.0)	20 (80.0)	14 (58.3)	N/A	20 (54.1)	22 (75.9)	13 (50.0)
After 12 weeks	12 (21.8)	40 (46.0)	5 (20.0)	10 (41.7)		17 (46.0)	7 (24.1)	13 (50.0)
Proportion of women with hyperglycaemia in pregnancy seen for pre-pregnancy counselling								
0-20%	110 (80.9)	55 (68.8)	21 (84.0)	17 (58.6)	67 (82.7)	23 (76.7)	21 (72.4)	15 (71.4)
21-40%	16 (11.8)	16 (20)	1 (4.0)	6 (20.7)	11 (13.6)	4 (13.3)	4 (13.8)	6 (28.6)

41-60%	5 (3.7)	5 (6.3)	2 (8.0)	3 (10.3)	0 (0.0)	2 (6.7)	3 (10.3)	0 (0.0)
61-80%	4 (2.9)	4 (5.0)	1 (4.0)	3 (10.3)	2 (2.5)	1 (3.3)	1 (3.5)	0 (0.0)
81-100%	1 (0.7)	0 (0)	0 (0.0)	0 (0.0)	1 (1.2)	0 (0.0)	0 (0.0)	0 (0.0)
Made changes to own practice in caring for women with hyperglycaemia in pregnancy over the previous three years+	N/A		N/A		N/A		N/A	
Yes		59 (64.1)		16 (61.5)		24 (70.6)		19 (59.4)
No		33 (35.9)		10 (38.5)		10 (29.4)		13 (40.6)
**Systems Indicators**								
	All regions		Central Australia		Far North Queensland		Top End	
	Baseline N (%)	Final N (%)	Baseline N (%)	Final N (%)	Baseline N (%)	Final N (%)	Baseline N (%)	Final N (%)
Perception of being well-supported in providing care for women with hyperglycaemia in pregnancy+	N/A		N/A		N/A		N/A	
Yes		88 (77.9)		27 (79.4)		31 (75.6)		30 (79.0)
No		11 (9.7)		4 (11.8)		3 (7.3)		4 (10.5)
Unsure		14 (12.4)		3 (8.8)		7 (17.1)		4 (10.5)
Perception of change in support in providing care for women with hyperglycaemia in pregnancy+	N/A		N/A		N/A		N/A	
Improved		65 (57.5)		22 (64.7)		22 (53.7)		21 (55.3)
Worsened		5 (4.4)		1 (2.9)		4 (9.8)		0 (0.0)
No change		11 (9.7)		2 (5.9)		8 (19.5)		1 (2.6)
Unsure		32 (28.3)		9 (26.5)		7 (17.1)		16 (42.1)
**Health Practitioner Confidence**								
	All regions		Central Australia		Far North Queensland		Top End	
	Baseline N (%)	Final N (%)	Baseline N (%)	Final N (%)	Baseline N (%)	Final N (%)	Baseline N (%)	Final N (%)
Confidence to manage women with hyperglycaemia in pregnancy								
Very confident/confident	107 (60.1)	86 (72.3)	19 (54.3)	28 (75.7)	58 (58.6)	31 (73.8)	29 (67.4)	27 (67.5)
Neutral	52 (29.2)	24 (20.2)	8 (22.9)	7 (18.9)	35 (35.4)	7 (16.7)	9 (20.9)	10 (25.0)
Not confident/not at all confident	19 (10.7)	5 (4.2)	8 (22.9)	1 (2.7)	6 (6.1)	1 (2.4)	5 (11.6)	3 (7.5)
N/A	N/A^	4 (3.4)	N/A	1 (2.7)	N/A	3 (7.1)	N/A	0 (0.0)
Confidence providing postpartum care for women with a history of hyperglycaemia in pregnancy*								

Very confident/confident	33 (56.9)	89 (74.8)	15 (60.0)	27 (73.0)		34 (79.1)	17 (53.1)	28 (71.8)
Neutral	18 (31.0)	18 (15.1)	4 (16.0)	6 (16.2)	N/A	5 (11.6)	14 (43.8)	7 (18.0)
Not confident/not at all confident	7 (12.1)	4 (3.4)	6 (24.0)	2 (5.4)		1 (2.3)	1 (3.1)	1 (2.6)
N/A	N/A^	8 (6.7)	N/A	2 (5.4)		3 (7.0)	N/A	3 (7.8)

*Data for NT only as not asked in baseline survey in FNQ ^N/A not a response option in baseline survey #Excluded dietitian, diabetes educator, manager (occupations where >50% responded N/A in final survey) ~Excluded dietitian, medical specialist (occupations where >50% responded N/A in final survey) +Asked only in final survey
